# The Anti-Serotonin Effect of Parthenolide Derivatives and Standardised Extract from the Leaves of *Stizolophus balsamita*

**DOI:** 10.3390/molecules24224131

**Published:** 2019-11-15

**Authors:** Joanna Nawrot, Marta Napierała, Kinga Kaczerowska-Pietrzak, Ewa Florek, Justyna Gornowicz-Porowska, Ewa Pelant, Gerard Nowak

**Affiliations:** 1 Department of Medicinal and Cosmetic Natural Products, Poznan University of Medicinal Sciences, Mazowiecka 33, 60-623 Poznan, Poland; joannac@ump.edu.pl (J.N.); kingak.pietrzak@gmail.com (K.K.-P.); justynagornowicz1@poczta.onet.pl (J.G.-P.); ewalawniczak@ump.edu.pl (E.P.); 2 Laboratory of Environmental Research, Department of Toxicology, Poznan University of Medicinal Sciences, Dojazd 30, 60-631 Poznan, Poland; martan@ump.edu.pl (M.N.); eflorek@ump.edu.pl (E.F.)

**Keywords:** antiserotonin effect, compositae, *Stizolophus balsamita*, parthenolide derivatives

## Abstract

The presence of dominant active compounds in standardised methanol extract from the leaves of *Stizolophus balsamita* (*S. balsamita*) was examined using HPLC with a diode-array detector. The extract and three dominant parthenolide derivatives were tested with Serotonin Research ELISA for their ability to inhibit the serotonin release from platelets. The antiserotonin effect of the extract was compared with that of parthenolide, a compound with proven antiserotonin and antimigraine effects. This study aimed to evaluate the ability of natural parthenolide derivatives to inhibit serotonin release from platelets. Izospiciformin, stizolin and stizolicin were analysed along with the standardised alcohol extract of *S. balsamita* leaves, which also contained four other parthenolide derivatives. All the analysed substances were found to inhibit serotonin release from platelets as compared with the control sample, which had 100% of serotonin released. Izospiciformin had the most significant impact (97.98% serotonin release inhibition). The effect of the methanol extract of *S. balsamita* on the serotonin release inhibition was also statistically significant.

## 1. Introduction

**Figure 1:**
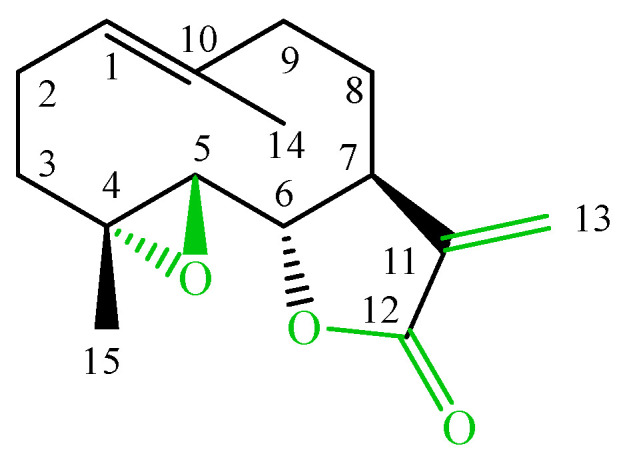
The structure of parthenolide.

Sesquiterpene lactones are commonly identified in many compositae genera and often highlighted as reliable chemosystematic markers [1]. The lactones exert numerous biological activities, e.g., anti-inflammatory, gastroprotective, antibacterial and antimigraine activities [2]. Particularly interesting are germacranolides—parthenolide (Figure 1) and its derivatives, with three elements in the skeleton: 4,5-epoxide and a lactone ring coupled with an exomethylene, which enables inhibition of cellular enzymes through the Michael nucleophilic addition [3].

Serotonin (5-HT) is a key molecule in the neurobiology of migraine. It is known that an initial high concentration of serotonin can cause vascular smooth muscle contraction and contribute to migraine aura formation [4]. Low serotonin concentrations, on the other hand, may stimulate perivascular pain fibres by their effect on the release of NO, prostaglandin and other factors responsible for vasodilation activity [5].

Serotonin is stored in enterochromaffin cells located in the lining of the gut (90%), platelets and serotonergic neurons of the central nervous system (CNS). Serotonin acts as a neurotransmitter in the CNS and as an autacoid in the nervous circuit—A local hormone with strong vasoconstrictor properties. Serotonin affects the nervous system by activating specific receptors with 5-HT_1B_ (located mostly in the brain vessels) and 5-HT_1D_ (found in the trigeminal nerve endings), playing a crucial part in migraine pathophysiology [6].

Migraine is a severe condition that affects people′s health and their socioeconomic situations. It is also a specific pharmacological puzzle, as its multitude of symptoms of unknown aetiology result in patients undergoing only symptomatic treatment.

Natural drugs may be an option for causative migraine treatment. *Tanacetum parthenium* (*T. parthenium*), known as feverfew, is a well-studied plant with proven antimigraine properties; the main active compound present in this plant is a parthenolide [7].

The antimigraine effect of parthenolide results from the modification of biochemical pathways, that is, the release of serotonin from platelets is inhibited [8], and the release of neuronal serotonin is reduced without significant effects on the 5-HT_2A_ and 5-HT_2B_ receptors [9].

The extract of *T. parthenium* herb enriched with parthenolide has been found to significantly reduce nitroglycerin-induced Fos expression in the nucleus trigeminalis caudalis. Purified parthenolide has been shown to significantly inhibit nitroglycerin-induced neuronal activation in additional brain nuclei and activity of nuclear factor-kappa B. These findings strongly suggest that parthenolide might be the component responsible for the biological activity of *T. parthenium* owing to its antimigraine effect [10]. Unfortunately, the studies showed that the concentration of parthenolide in *T. parthenium* decreases significantly (13%) during storage [11].

Another study showed that some phenolic compounds might also be responsible for the antimigraine effect of *T. parthenium*. These compounds reduce lactate dehydrogenase (LDH) release, nitrite levels and 5-HT, and thus, they cause antioxidant and antiapoptotic effects [12].

The phytochemical profiles of plants in genus *Stizolophus* show the presence of parthenolide derivatives [1,13], and in their structures, all the elements responsible for the parthenolide activity were observed. For this study, *Stizolophus balsamita* (*S. balsamita*) (Lam.) K.Koch from Iran was chosen, as no less than seven parthenolide derivatives were found in it [14].

**Figure 2:**
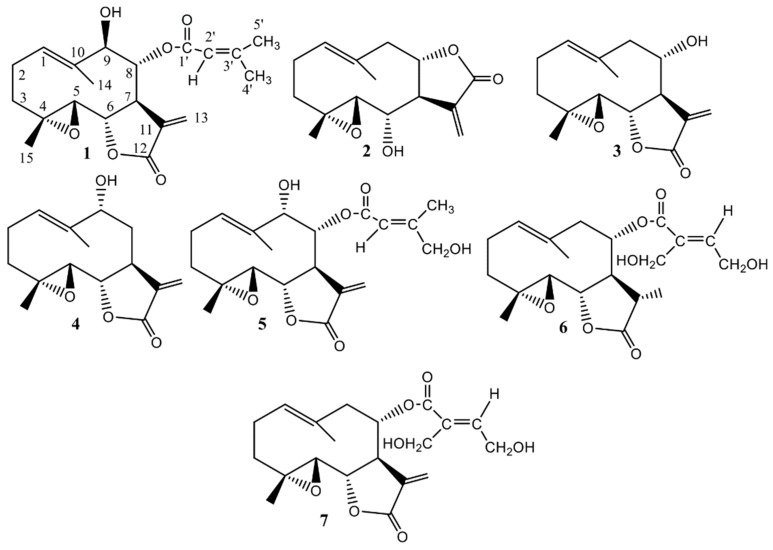
Chemical structures of compounds isolated from the leaves of *Stizolophus balsamita* (*S. balsamita*).

The presence of parthenolide derivatives in *S. balsamita*, including balsamin (**1**), izospiciformin (**2**), stizolin (**3**), 9α-hydroxyparthenolide (**4**), 8α-*E-*(4′-hydroxy)-senecioyloxy-9α-hydroxyparthenolide (**5**), 11βH,13-dihydrostizolicin (**6**) and stizolicin (**7**) [14,15] (Figure 2), induces authors to test for antiserotonin properties of the three germacranolides—**2**, **3**, **7**—and the methanol extract isolated from the leaves of *S. balsamita*. Our research shows that through the thin-layer chromatography method a detailed description of the chemical structure of parthenolide derivatives may be obtained (Figure S1).

## 2. Results and Discussion

**Figure 3:**
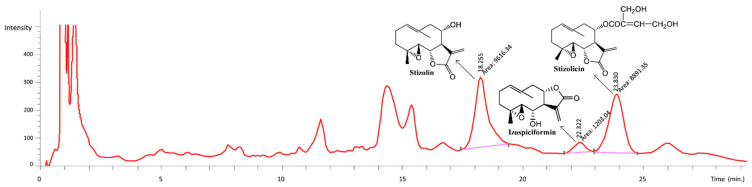
The HPLC chromatogram of the methanol extract from the *S. balsamita* leaves.

The amounts of characteristic compounds **2**, **3** and **7** in the methanol extract from the leaves of *S. balsamita* was determined using HPLC (Figure 3).

The amounts of the three compounds are as follows: compound **2**, 0.04 mg/mL (2.22 mg/g of the extract); compound **3**, 0.44 mg/mL (22.05 mg/g of the extract); compound **7**, 0.33 mg/mL (16.5 mg/g of the extract). The concentrations of these compounds were all 40.77mg/g (4.08%). More importantly, the concentrations of the active compounds in *S. balsamita* leaves did not change over a considerable time (no measurable decrease for at least three years), and their pharmacological activities were constant (Figure S2).

**Figure 4:**
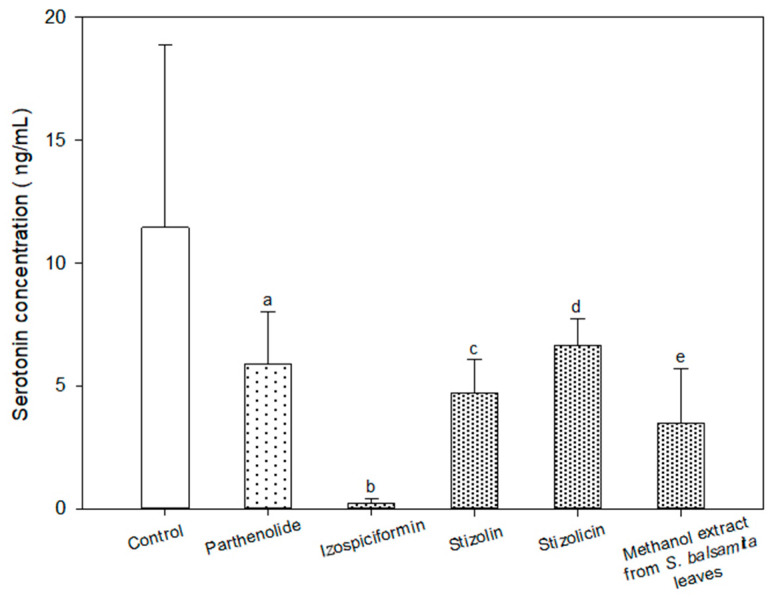
Serotonin concentration (average ± SD; ng/mL) in the sample after the administration of analysed substances: parthenolide (a), izospiciformin (b), stizolin (c), stizolicin (d) and methanol extract from the *S. balsamita* leaves (e). a—statistically significant difference compared to the control sample (*p* = 0.0477); b—statistically significant difference compared to the control sample (*p* = 0.0001); c—statistically significant difference compared to the control sample (*p* = 0.0380); d—statistically significant difference compared to the control sample (*p* = 0.0389); e—statistically significant difference compared to the control sample (*p* = 0.0097).

The concentration of serotonin (ng/mL) in the samples containing parthenolide, compounds **2**, **3** and **6** and in the methanol extract of the *S. balsamita* leaves were significantly lower than that in the control sample (Table 1 and Figure 4, Figures S3–S6).

One can assume that the mechanism behind such an effect of the analysed compounds and the methanol extract from the leaves of *S. balsamita* is analogical to that of the parthenolide. It is known that the parthenolide interacts with transient receptor potential ankyrin 1 (TRPA1) nucleophilic sites, which leads to the inhibition of nociception and neurogenic vasodilatation in the trigeminovascular system. Moreover, parthenolide and the related sesquiterpene lactones have been shown to inhibit the activation of the pro-inflammatory transcription factor, nuclear factor-kB (NF-kB) by different stimuli, such as phorbol esters, tumour necrosis factor-α and hydrogen peroxide [6]. The analysed compounds may inhibit serotonin release through its effect on protein kinase C (PKC). The activation of PKC by phorbol esters resulted in a reduction of serotonin uptake in endothelial cells and platelets cells. PKC is considered a critical regulator of central sensitization and is recognized to be involved in the pathogenesis of chronic migraine. [16]

## 3. Materials and Methods

### 3.1. Plant Material

Leaves of *S. balsamita* (Lam.) K.Koch (Compositae) were collected from the Botanical Garden at the Department of Medicinal and Cosmetic Natural Products, University of Medical Sciences in (Poznan, Poland), where the voucher specimens (voucher number: 42/2014) are deposited. Seeds of *S. balsamita* were provided by the Botanical Garden in (Teheran, Iran). Flowers, leaves and seeds of *S. balsamita* were identified by Prof. Karol Latowski from Adam Mickiewicz University in Poznan.

### 3.2. Extraction, Isolation and Identification of Compounds from S. balsamita Leaves

From the leaves of this species, seven germacranolides (parthenolide derivatives) were isolated and identified: balsamin (**1**), izospiciformin (**2**), stizolin (**3**), 9α-hydroxyparthenolide (**4**), 8α-*E-*(4′-hydroxy)-senecioyloxy-9α-hydroxyparthenolide (**5**), 11βH,13-dihydrostizolicin (**6**) and stizolicin (**7**). The data on the isolation and structural elucidation of compounds **1**–**7** are described in detail in our previous work [14].

The compounds were separated by column chromatography on the silica gel (particle size: 0.063–0.200 mm; Merck [Darmstadt, Germany] Art. 7733). Selected fractions were further rechromatographed on the silica gel with particle sizes of <0.063 mm (Merck Art. 7729). The NMR spectra were run on a Bruker Avance 600 instrument using 600 and 150 MHz frequencies for hydrogen nuclei (^1^H) and carbon nuclei (^13^C), respectively, and tetramethylsilane (TMS) was used as an internal standard. The spectra were obtained for CDCl_3_ or DMSO-*d_6_* solutions at 298 K. It should be emphasized that NMR measurements were made in aprotic solvents without the addition of solvents causing deuterium exchange, like CD_3_OD, D_2_O or TFA-_d_ (CF_3_COOD), but containing residual water, which is crucial for the hydroxyl group detection in the NOESY spectra. Therefore, those solvents must not be dried to remove the mentioned water completely. Chemical shifts δ are given in ppm, and coupling constants *J* are given in Hz [14,15].

Parthenolide did not occur in the analysed plant. This compound was bought from the Sigma-Aldrich company (Saint Louis, MO, USA) and was used for the comparative analysis.

### 3.3. HPLC Chromatography Analysis

The concentrations of dominant compounds in the methanol extract of *S. balsamita* leaves were determined by HPLC. The analysis was conducted using an Agilent 1200 SL liquid chromatography device with a diode detector and an HPLC precolumn LiChrospher 60 RP-Select B (5 µm, 125-4) (Merck). Data and chromatograms were gathered using ChemStation software for the LC 3D system revision B.04.01 SP1 (Agilent Technologies) (Santa Clara, CA, USA). Further analysis was conducted with ChemStation for the LC 3D system revision B.04.01 SP1, Microsoft Excel 2000 (Redmond, WA, USA) and Sigmaplot 11-11.0.0.77.) (Chicago, IL, USA). The mobile phase was composed of water and methanol, and the following gradient elution method was used: 5% methanol for 0 min; 30% methanol for 12 min; 30% methanol for 25 min; and 50% methanol for 30 min. During this process, the phase flow rate was constant and reached 1 cm^3^/min. The volume of the dispensed sample was 15 µL. Absorbance was measured at *λ* = 242 nm.

### 3.4. Biological Material

Ten female Wistar albino rats (approximately 200 g body weight) were obtained from the Laboratory Animals Breeding Center, Department of Toxicology, Poznan University of Medical Sciences, Poznan, Poland. The Local Ethics Committee for Animal Research (Poznan, Poland) approved the collection of the biological material (resolution no. 20/2012 from 11 May 2012). The research was conducted in the years 2016 and 2017.

Blood samples were withdrawn from the heart chambers (8 mL from each animal) and collected into trisodium tubes at a ratio of 1:9 (*v*/*v*). After centrifugation at 160× *g* for 10 min followed by 500× *g* for 10 min at 37 °C, the platelet-rich plasma (PRP) was obtained with a mean number of platelets of 1119 + [10^3^/µL].

Methanol solutions of the dominant compounds **2**, **3** and **7** were added to the PRP along with the methanol extract from the *S. balsamita* leaves. The methanol parthenolide solution was used as a reference, and a solution of 0.9% NaCl and ammonium dihydrogen orthophosphate (ADP) was used as an aggregation inducer.

### 3.5. Enzyme-Linked Immunosorbent Assay (ELISA)

Ultrasensitive enzyme immunoassay (Serotonin Research ELISA BA E-5937 by Labor Diagnostika Nord GmbH & Co. KG. (Nordhorn, Germany) was used for the quantitative determination of serotonin. The results were obtained by comparing the absorbance measured from the standard curve prepared using standard samples according to the manufacturer’s specification. The analysed compounds and the dry extract were dissolved in 95% ethanol to obtain solutions with a 20 mg/mL concentration. Further, 30 µL of each solution was washed in 970 µL phosphate-buffered saline (PBS). Each sample had a concentration of 0.6 ng/mL. ADP was dissolved in 0.9% NaCl, and a solution with a concentration of 1 mM/L was obtained. Four hundred and sixty microlitres of PRP was mixed with 100 µL of the extract or other compound solutions for two minutes at 37 °C at 1000 rpm. Two control samples were prepared: K1 and K2, each containing 460 µL PRP with 100 µL 3% EtOH dissolved in PBS.

### 3.6. Statistical Analysis

The analysed data were not normally distributed (the Shapiro–Wilk test). A comparison of the examined compounds was conducted with the Mann–Whitney U test. The results are displayed as average ± standard deviation. All statistical analyses were performed using Statistica 12 PL (StatSoft) (Round Rock, TX, USA). All the tests were considered significant at *p* < 0.05.

## 4. Conclusions

All the analysed substances (compounds **2**, **3** and **7** and the methanol extract from *S. balsamita* leaves) were found to inhibit the release of serotonin from platelets, and the results were statistically significant. Compound **2** had the most substantial effect, which may be caused by the different structure of its germacranolide skeleton as compared to the other analysed lactones. In compound **2**, the lactone ring is in position 7–8, not position 6–7 as in compounds **3** and **7** and other parthenolide derivatives.

Compounds **2** and **3** and the methanol extract of the *S. balsamita* leaves showed statistically significant higher biological activities as compared to parthenolide. The difference in the structure between the parthenolide and its derivatives may be stressed. The results showed that the additional element (substituent on C-8) in the parthenolide structure strengthens the pharmacological effect of the compounds.

## Figures and Tables

**Table 1:** The concentration of serotonin in the samples with the analysed substances and in the control samples.

N	Sample	Average Serotonin Concentration (ng/mL)	Standard Deviation
12	Control	11.44	7.42
12	Parthenolide	5.90	2.11
9	Izospiciformin	0.23	0.19
12	Stizolin	4.72	1.36
12	Stizolicin	6.62	1.08
12	Methanol extract from the *S. balsamita* leaves	3.48	2.21

N = number of valid results.

## Supplementary Materials

The following are available online—Figure S1: TLC (thin-layer chromatography) of the purified CH_2_Cl_2_ extract and germacranolides isolated from the *S. balsamita* leaves; Figure S2: HPLC chromatograms of stizolin (S), izospiciformin (I) and stizolicin (Sc); Figure S3: Concentrations of serotonin (unit: ng/mL) in the stizolin sample and the control sample; Figure S4. Concentrations of serotonin (unit: ng/mL) in the izospiciformin sample and the control sample; Figure S5. Concentrations of serotonin (unit: ng/mL) in the stizolicin sample and the control sample; Figure S6. Concentrations of serotonin (unit: ng/mL) in the methanol extract from the leaves of *S. balsamita* sample and the control sample.
