# Correction: Nawrot et al. The Anti-Serotonin Effect of Parthenolide Derivatives and Standardised Extract from the Leaves of *Stizolophus balsamita*. *Molecules* 2019, *24*, 4131

**DOI:** 10.3390/molecules30071428

**Published:** 2025-03-24

**Authors:** Joanna Nawrot, Marta Napierała, Kinga Kaczerowska-Pietrzak, Ewa Florek, Justyna Gornowicz-Porowska, Ewa Pelant, Gerard Nowak

**Affiliations:** 1 Department of Medicinal and Cosmetic Natural Products, Poznan University of Medicinal Sciences, Mazowiecka 33, 60-623 Poznan, Poland; 2 Laboratory of Environmental Research, Department of Toxicology, Poznan University of Medicinal Sciences, Dojazd 30, 60-631 Poznan, Poland

The authors would like to make the following correction to the published paper [1]. We have deleted the detailed information regarding the procedure of isolating and identifying germacranolides from *S. balsamita*, which was included in Section 3.2 and the Supplementary Files (Tables S1 and S2) and refer to the data in our previous paper [14] (in the original publication). We have added the following sentence at the end of the first paragraph in Section 3.2:

The data on the isolation and structural elucidation of compounds **1**–**7** are described in detail in our previous work [14].

The authors state that the scientific conclusions are unaffected. This correction was approved by the Academic Editor. The original publication has also been updated.
